# Composite Injection Technique With a Digital Workflow: A Pragmatic Approach for a Protruding Central Incisor Restoration

**DOI:** 10.7759/cureus.58712

**Published:** 2024-04-22

**Authors:** Diana F Muslimah, Yuichi Hasegawa, Tichy Antonin, Foxton Richard, Keiichi Hosaka

**Affiliations:** 1 Department of Regenerative Dental Medicine, Graduate School of Biomedical Sciences, Tokushima University, Tokushima, JPN; 2 Department of Dental Laboratory, Dental Hospital, Tokyo Medical and Dental University, Tokyo, JPN; 3 Department of Dentistry, Institute of Dental Medicine, First Faculty of Medicine of the Charles University and General University Hospital, Prague, CZE; 4 Division of Conservative Dentistry, King’s College London Dental Institute at Guy’s, King’s and St Thomas’ Hospitals, King’s College London, London, GBR; 5 Division of Interdisciplinary Research for Medicine and Photonics, Institute of Post-LED Photonics, Tokushima University, Tokushima, JPN; 6 Microsystems Technology Laboratories, Massachusetts Institute of Technology, Cambridge, GBR

**Keywords:** pragmatic esthetics, cut-back technique, protruding maxillary central incisor, digital workflow, direct composite injection technique

## Abstract

Esthetic concerns frequently drive individuals to seek dental treatment, leading to a rising demand for minimally invasive and time-efficient procedures. The Minimal Intervention Dentistry (MID) concept, which employs dental adhesive and resin composites, offers an effective approach for enhancing esthetics while preserving natural tooth structure. This case report outlines the esthetic enhancement of a protruding maxillary right central incisor through a direct composite restoration approach, utilizing a composite injection technique with a digital workflow. A 42-year-old male patient presented with a discolored and protruding maxillary right central incisor. After declining orthodontic treatment due to time and cost constraints, the patient opted for an alternative approach. A digital wax-up was 3D printed to create a clear silicone index, enabling precise resin composite injection to achieve the desired esthetic outcome. After internal bleaching and minimal labial surface reduction, a flowable resin composite was applied freehand to the mesial-proximal surface using a curved plastic matrix manufactured universally. The composite injection technique was subsequently employed through the incisal opening of the clear silicone index to shape the labial surface and incisal edge. In order to address insufficient tooth reduction, an additional partial labial resin composite cutback was performed, ensuring minimal reduction while enhancing esthetics. This direct composite veneer restoration, combining conventional proximal surface creation with a composite injection technique utilizing a custom-made clear silicone index prepared via a digital workflow, emerged as a pragmatic solution in a case where orthodontic treatment was not preferred. Moreover, in situations of insufficient dentin preparation, additional cutback preparation and composite placement can enhance color matching with minimal reduction. The direct composite restoration, facilitated by the composite injection technique and digital workflow, effectively rectified the inclination of the protruding maxillary central incisor, highlighting the potential of this approach in addressing esthetic dental concerns. The research and clinical technique presented in this case report hold clinical importance by offering a minimally invasive and practical alternative to orthodontic treatment and conventional restorations for patients with esthetic concerns. The composite injection technique with a digital workflow preserves natural tooth structure, reduces chair time, and enhances esthetic outcomes. This approach is particularly relevant to esthetic dentistry as it addresses anterior dental malalignment and discolored teeth while prioritizing patient satisfaction and individualized care, aligning with the principles of pragmatic esthetics and MID. The potential for long-term durability and patient satisfaction makes it a valuable addition to esthetic dental practice.

## Introduction

Pragmatic esthetics prioritizes practical considerations and patient well-being over elusive ideals of perfection in dental treatment [1]. The Minimal Intervention Dentistry (MID) concept has gained attention for its effective approach to addressing esthetic concerns through dental adhesive and resin composites [2]. MID emphasizes the preservation of tooth structure while achieving favorable esthetic outcomes, applied in various cases such as space closure [3], recontouring crown morphology [4], anterior tooth prostheses [5], and correction of dental malalignment [3].

Conventionally, bonded porcelain restorations have served as an alternative to orthodontic treatment for correcting anterior dental malalignment [6]. Despite their effectiveness, these indirect restorations are more invasive, time-consuming, and costly compared to direct restorations. Moreover, indirect restorations that involve tooth preparation can expose a significant amount of dentin, necessitating a careful selection of restoration techniques. The resin coating technique, developed in the early 1990s, involves applying a resin layer to exposed dentin prior to placing an indirect restoration, thereby enhancing the bond strength [7]. However, Akehashi et al. found that direct bonding to dentin can achieve higher dentin bond strengths with appropriate adhesive materials [8].

On the other hand, direct free-hand composite restorations can be technically challenging, often leading practitioners to opt for more invasive indirect restorations, especially in anterior esthetic cases where restoration morphology significantly impacts esthetics [3]. To address this challenge, an innovative approach involving the injection of flowable resin composite into a prefabricated clear silicone index has emerged, offering a promising way of simplifying and expediting direct restorations [4]. This composite injection technique, utilizing a clear index as a mold, has demonstrated its potential in reducing chair time and delivering predictable outcomes, although it requires prior laboratory preparation. Furthermore, with recent advancements in digital technology, laboratory work can be elevated to a higher level of precision and reliability, enabling a seamless transition from the digital wax-up to the final restorative form [3,9-15].

Nevertheless, the nature of the injection technique itself may result in some overflow beyond the margin or pose challenges in creating an appropriate proximal contact. Therefore, there may arise a need for the incorporation of conventional proximal surface creation techniques, such as the use of a plastic matrix [16], in conjunction with the injection technique to effectively address these challenges. This approach not only addresses potential overflow challenges but also enhances the precision and esthetics of the proximal contact, thereby contributing to the overall success of the dental restoration process.

In light of these considerations, this case report presents the application of the aforementioned techniques within a minimally invasive framework to enhance the esthetics of a protruding, discolored maxillary right incisor. This patient, who declined orthodontic treatment due to time constraints and cost considerations, benefited from the utilization of a digital wax-up and a clear silicone index, which enabled precise composite injection molding. The incorporation of the cutback technique and conventional proximal surface creation further optimized esthetic outcomes while preserving valuable tooth structure.

## Case presentation

**Figure 1:**
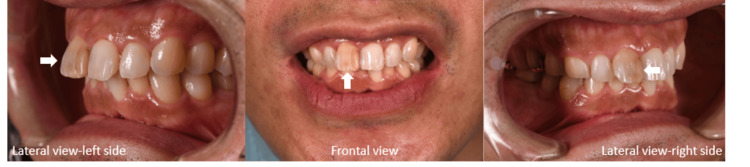
Preoperative view of a protruded maxillary right central incisor.

A 42-year-old male patient sought treatment to address the esthetic concerns related to his maxillary right central incisor. The tooth was protruded and discolored as a result of previous endodontic treatment (Figure 1). While initially considering orthodontic treatment, the patient ultimately opted for a more conservative approach due to time, cost constraints, and an upcoming overseas relocation. After receiving comprehensive information about the proposed treatment, the patient gave informed consent.

Internal bleaching and preparation of a clear index for composite injection technique with a digital workflow

**Figure 2:**
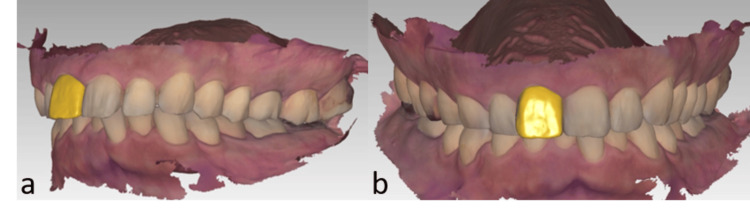
Digital wax-up in lateral view (a) and frontal view (b).

During the initial appointment, internal bleaching was performed to improve the tooth's color and achieve a uniform shade. Simultaneously, a digital impression was obtained using an intraoral scanner (Trios3, 3Shape A/S, Copenhagen, Denmark). A digital wax-up was created using a CAD application (3Shape Dental Systems, 3Shape A/S) to simulate the desired final outcome, with the goal of correcting the tooth's protrusion (Figure 2a, b). The digital wax-up was created with consideration of minimal preparation of the teeth and to obtain the golden proportion as close as possible.

**Figure 3:**
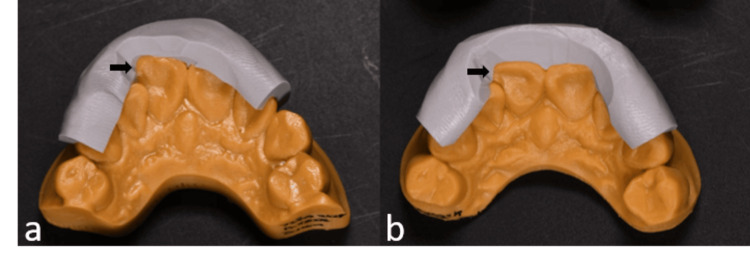
3D printed models of initial dentition (a) and digital wax-up model (b) and prepared silicone guides for each model.

**Figure 4:**
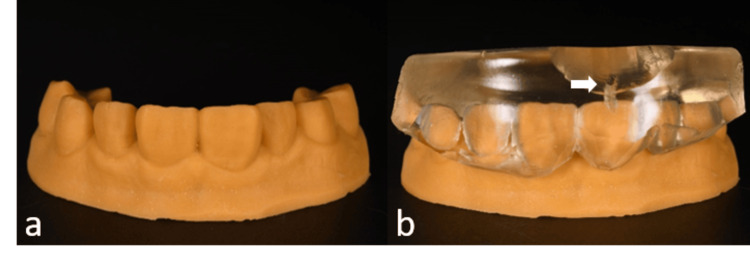
A 3D printed model generated from digital waxing (a). A clear silicone index with the trimmed entrance of an opening to facilitate easy composite injection (b).

3D printed models were generated to represent the initial dentition (Figure 3a) and the simulated desired outcome (Figure 3b). These models were used to create silicone keys to determine the appropriate preparation thickness. Additionally, the wax-up model (Figure 4a) served as the basis for creating a clear silicone index for the injectable resin composite technique (Figure 4b). The index was fabricated using clear polyvinyl siloxane material (EXACLEAR, GC Corp., Tokyo, Japan) and polymerized at +0.2 MPa pressure for 10 minutes to prevent air bubble formation. An incisal opening was prepared on the silicone index (Figure 4b).

Composite placement with/without the clear silicon index and cutback technique

**Figure 5:**
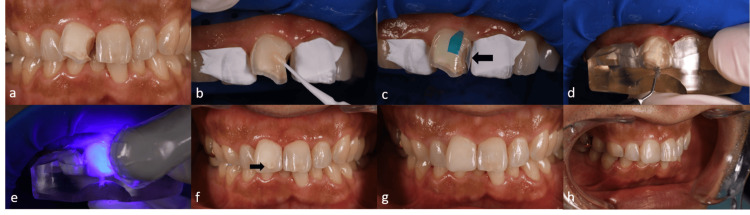
After removing the old restoration and approximately 1.5 mm reduction, the dentin surfaces were exposed (a). A two-step self-etching adhesive system was applied (b). The mesial surface was created before the composite injection technique to make appropriate proximal contact (c). Inverse injection of flowable resin composite through the occlusal opening of a clear silicone index (d). Polymerization was performed (e). The dentin was visible after the index was removed due to insufficient reduction (f). The dentin was cut back, and an additional flowable composite was placed (g). Lateral view of the final restoration (h).

At the subsequent appointment, the tooth displayed a successful result after bleaching (Figure 5a). The discolored restoration was removed, and precise tooth preparation was conducted using silicone keys based on the initial and post-restoration models (Figure 5b). The tooth was isolated using a split rubber dam technique and cleaned with pumice and a chemical cleaning agent (Katana Cleaner, Kuraray Noritake Dental, Japan). A two-step self-etch adhesive system (Clearfil SE Bond 2, Kuraray Noritake Dental) was applied with selective enamel etching using phosphoric acid gel (K-Etchant Syringe, Kuraray Noritake Dental) following the manufacturer’s instructions (Figure 5c). To achieve an ideal interproximal contact, a sectional curved plastic matrix (Sectional matrix, Kerr) was positioned (Figure 5d), and a flowable resin composite (Clearfil ES Flow Universal, U shade, Kuraray Noritake Dental Inc.) was placed on the proximal surface (Figure 5e). The clear silicone index was then seated, and the same flowable resin composite was inversely injected through the incisal opening of the clear silicone index (Figure 5f) and polymerized using an LED light curing unit (PenCure 2000, J. Morita, Tokyo, Japan) (Figure 5g). Upon removal of the index, exposed dentin was observed on the labial surface, indicating insufficient reduction depth. To correct this, the exposed dentin was reduced (ca. 1.5 mm), and an additional layer of flowable resin composite (Clearfil ES Flow Universal, U shade, Kuraray Noritake Dental Inc.) was placed and polymerized following the two-step self-etch adhesive system, ensuring “truly” minimal dentin reduction.

Finishing and polishing of the restoration

The excess composite was removed using a #12 scalpel, followed by polishing procedures employing a two-step polishing system (FP9769M, FP9769F, Meisinger Polisher, Hager & Meisinger GmbH, Neuss, Germany) and a polishing particle-embedded brush (OptiShine™, Kerr, Brea, CA, USA). The injection technique allowed for minimal adjustments and significantly reduced chair-side finishing and polishing time (Figure 5h). After three years, the prognosis remained excellent. The patient provided informed consent for the publication of this case.

## Discussion

Esthetics stands as a primary concern motivating individuals to seek dental treatments, and this case report demonstrates the effectiveness of the composite injection technique within a digital workflow in achieving enhanced dental esthetics. Restoration using direct composite resin is a compelling alternative when constraints related to time and cost deter orthodontic treatment. This strategy aligns seamlessly with the principles of pragmatic esthetics, which prioritize practical considerations and patient satisfaction over theoretical ideals of dental perfection [1].

While the composite injection technique necessitates multiple patient visits due to the laboratory manufacturing process of the clear index, it simplifies and streamlines restoration placement compared to freehand direct composite restorations, which can be challenging and time-consuming chairside [17]. The approach is not only minimally invasive [4] and time-saving [17] but also offers superior bond durability to dentin compared to indirect bonding methods [8,18]. When choosing between direct and indirect composite resin restorations, the potential for dentin exposure underscores the importance of meticulous preparation and bonding techniques, as direct bonding to dentin exhibits superior performance to indirect bonding methods [8].

Concerns may arise regarding the longevity and mechanical properties of injectable resin composites when compared to ceramics [6]. However, the presented case demonstrates that a satisfactory clinical outcome in terms of esthetics can be achieved, aligning perfectly with the principles of pragmatic esthetics. The cutback technique minimizes tooth reduction and augments the overall esthetic result. This comprehensive approach meticulously considers the natural anatomy of the tooth, faithfully recreating it while preserving as much natural tooth structure as possible. Moreover, it effectively addresses issues like air voids that might occur due to the nature of flowable resin composites after the removal of the index or when minor tooth color enhancement using staining resin composites is necessary.

The amalgamation of the conventional creation of proximal surfaces with the injection technique overcomes the challenge of achieving optimal interproximal contact, ensuring a successful reconstruction of the labial surface and the inherent contour of the tooth. Although concerns about wear and fractures may arise with flowable resin composites, the ability to store and reuse the same silicone index can simplify future restoration repairs. Nevertheless, vigilant and regular monitoring of restorations remains vital to detect any complications, such as marginal discoloration or chipping [19], and to promptly address them and extend the longevity [20].

Incorporating digital workflows into this process enhances predictability, reduces technique sensitivity, and achieves a high level of accuracy in translating the visual representation of the desired outcome from the digital wax-up to the final form. As demonstrated by Gestakovski [4,14], this approach offers a multitude of benefits, including reduced chair time, predictable outcomes, and simplified maintenance compared to indirect restorations. However, further investigation is necessary to establish its long-term durability and clinical efficacy. Future research efforts, such as randomized controlled trials with larger sample sizes, are essential to validate these findings and assess long-term performance and patient satisfaction.

In summary, the restoration technique employed in this case has successfully addressed the patient’s esthetic requirements while adhering to the principles of minimally invasive dentistry and pragmatic esthetics. The selection of an appropriate restoration technique should take into account factors such as the specific case requirements, patient preferences, the expertise of the dentist, and the availability of materials. By considering these factors, dental professionals can offer individualized and effective solutions that prioritize patient satisfaction and overall dental well-being.

## Conclusions

In conclusion, the direct composite restoration approach, facilitated by the composite injection technique within a digital workflow, exhibits great potential for achieving optimal esthetic outcomes while preserving valuable tooth structure. Nonetheless, further research and comprehensive long-term clinical studies are imperative to unlock the full potential and durability of this approach in everyday clinical practice.
